# Increased Expression of Musashi-1 Evidences Mesenchymal Repair in Maxillary Sinus Floor Elevation

**DOI:** 10.1038/s41598-018-29908-3

**Published:** 2018-08-16

**Authors:** Francisco O’Valle, Juan G. de Buitrago, Pedro Hernández-Cortés, Miguel Padial-Molina, Vicente Crespo-Lora, Marien Cobo, David Aguilar, Pablo Galindo-Moreno

**Affiliations:** 1 Department of Pathology & Biopathology and Medicine Regenerative Institute (IBIMER, CIBM), University of Granada, Granada, Spain; 2 Institute of Biosanitary (Ibs-Granada), University of Granada, Granada, Spain; 3 Department of Oral Surgery and Implant Dentistry, School of Dentistry, University of Granada, Granada, Spain; 4 Department of Orthopedic Surgery, San Cecilio University Hospital of Granada, Granada, Spain; 5 Department of Genomic Medicine & GENYO, Centre for Genomics and Oncological Research, Pfizer – University of Granada – Andalusian Regional Government, Granada, Spain

## Abstract

This study aimed to analyze the expression of Musashi-1 (MSI1) in maxillary native bone and grafted bone after maxillary sinus floor elevation. To do so, fifty-seven bone biopsies from 45 participants were studied. Eighteen samples were collected from native bone while 39 were obtained 6 months after maxillary sinus grafting procedures. Musashi-1 was analyzed by immunohistochemistry and RT-PCR. MSI1 was detected in osteoblasts and osteocytes in 97.4% (38/39) of grafted areas. In native bone, MSI1 was detected in only 66.6% (12/18) of the biopsies, mainly in osteocytes. Detection of MSI1 was significantly higher in osteoprogenitor mesenchymal cells of grafted biopsies (p < 0.001) but minor in smooth muscle and endothelial cells; no expression was detected in adipocytes. The mesenchymal cells of the non-mineralized tissue of native bone showed very low nuclear expression of MSI1, in comparison to fusiform cells in grafted areas (0.28(0.13) vs. 2.10(0.14), respectively; p < 0.001). Additionally, the detection of *MSI1* mRNA was significantly higher in biopsies from grafted areas than those from native bone (1.00(0.51) vs. 60.34(35.2), respectively; p = 0.029). Thus, our results regardig the significantly higher detection of Musashi-1 in grafted sites than in native bone reflects its importance in the remodeling/repair events that occur after maxillary sinus floor elevation in humans.

## Introduction

Musashi-1 (gene *MSI1*) is a phylogenetically preserved RNA-binding protein first detected in the nervous system^1–3^. It was isolated as the homologous in mammals of the protein present in *Drosophila melanogaster*. MSI1 contains 362 aminoacids with two RNA-binding ribonucleoprotein motifs (RBD1 y RBD2)^4^. Genes encoding Musashi exert regulatory functions on progenitor cells of adults and developing organisms^5^. MSI1 is required for the asymmetric division of neuronal precursors^6,7^. It also maintains the self-renewal capacities of stem cells^8–10^.

The main function of MSI1 is to inhibit the translation of mRNA targets such as *ttk69* and *m-numb* that are key in the function of progenitor cells^9,11^. *m-numb* inhibits *Notch* signaling which is essential for maintaining self-renewal potential^12^. *m-numb* is silenced by MSI1 by binding to the 3′UTR of the mRNA encoding it^3^. This occurs simultaneously to the translational inhibition of cyclin-dependent kinase inhibitor 1 (or p21^WAF-1^), a regulator of the cell cycle^13^. Therefore, by both actions on *m-numb* and p21^WAF-1^, MSI1 un-inhibits *Notch* signaling, which in turn results in cell proliferation and differentiation^14^. Thus, MSI1 has been reported as a marker of mesenchymal stromal cells^15,16^.

Adult mesenchymal stromal cells (MSCs) are a group of pluripotent adherent fusiform cells with notable plasticity and potential to differentiate into osteoblasts, chondrocytes and adipocytes^17–20^. Among other tissues, MSCs can be obtained from adipose tissue, umbilical cord blood and bone marrow^21^. They are also known by other names, including marrow stromal cells, fibroblastoid colony forming units, stromal precursors or multi-potent adult progenitor cells (MAPCs).

Within the context of dental implantology, insufficient bone volume is a frequent challenge for the occlusal rehabilitation of the edentulous maxilla by implant supported prosthesis. Specifically, in the postero-superior region, bone volume is limited by the presence of the maxillary sinus. Fortunately, maxillary sinus floor elevation procedures can solve the problem with the use of bone grafts. The original technique^22^ and its modifications have reported high success rates. However, it is important to know the biological mechanisms that intervene in the healing process after grafts are placed in the sinus.

Bone repair is guided by the presence of MSCs with the potential to differentiate to osteoprogenitor cells in the grafted area. Thus, the aim of the current study was to analyze the expression of Musashi-1 in biopsies obtained from grafted posterior maxillary bone and to compare the results with those of native bone from non-grafted posterior maxillary bone.

## Material and Methods

### Study participants

The study was designed according to the STROBE guidelines for observational studies. For this cross-sectional study, all procedures were performed according to the principles of the Declaration of Helsinki and after obtaining informed consent. The Human Research Ethics Committee of the University of Granada approved the study protocol before any procedure was initiated (307/CEIH/2017).

To perform the study, bone samples were collected from partially or totally edentulous patients admited at a Dental Faculty practice of the University of Granada in need of tooth replacement in the posterior maxillary region by dental implants with less than 5 mm of initial residual crestal bone who had been subjected to maxillary sinus floor elevation 6 months earlier. The procedure had to have been performed following a routine protocol established in our clinic. Briefly, under local anesthesia (Ultracain®, Laboratorios Normon, S.A., Madrid, Spain), after flap elevation, access to the sinus was achieved by a bone scraper that allows the collection of autogenous bone (Safescraper®, Meta, Reggio Emilia, Italy). The Schneiderian membrane was then carefully elevated and the sinus cavity was grafted using a 1:1 mix of the autogenous bone collected with the bone scraper (ACB) and anorganic bovine bone (ABB) particles (Geistlich Bio-Oss® - Geistlich Pharma AG, Wolhusen, Switzerland). In all cases, the lateral aspect of the bony window was covered with an absorbable collagen membrane (Geistlich Bio-Gide® - Geistlich Pharma AG). Primary wound closure was achieved in all cases. All participants were prescribed with 875 mg of amoxicillin combined with 125 mg of clavulanic acid every 8 hours for 7 days post-surgery and 600 mg of ibuprofen tablets as needed, to a maximum of 3600 mg per day. Sutures were removed two weeks after sinus floor elevation.

Additionally, biopsies were collected from adjacent areas if implants were also to be placed as part of the prosthetic plan.

### Histopathological study

To quantify the percentage of new mineralized tissue, remnant ABB particles (only in grafted bone) and non-mineralized tissue, bone biopsies were collected by using 3 mm diameter trephines from the location were the implants would be placed. They were then immediately fixed in 10% formalin for 48 h at 4 °C. They were then decalcified during 24 h at 37 °C (Decalcifier I^®^, Surgipath Europe Ltd., Peterborough, United Kingdom) and embedded in paraffin. 4 µm sections were obtained following the apico-coronal axis, deparaffinized, rehydrated and stained with hematoxylin-eosin, PAS and Masson’s trichrome. By using the 40x objective in a microscope with an attached scale (BH2, Olympus Optical Company, Ltd., Tokyo, Japan), the number of osteoblasts, osteoclasts, osteocytes and vessels were quantified per mm^2^.

Histomorphometric quantification was performed semiautomatically using the Masson’s trichrome stain. 10 random images were captured from each sample with a 10x objective in a microscope with a digital camera attached (DP70, Olympus Optical Company). Images were then analyzed with the software ImageJ (NIH, http://imagej.nih.gov/ij/).

### Immunohistochemical analysis

Expression of Musashi-1 was evaluated semiquantitatively by a 0–3 scale (0 = no staining; 1 = weak; 2 = moderate; 3 = intense) in osteocytes, osteoblasts, osteoclasts, epithelial cells in the Schneiderian membrane, mesenchymal cells, fibroblasts, smooth muscle cells, endothelial cells and adipocytes. To do so, rehydrated samples were also termically treated in a pre-treatment module (Thermo Fisher Scientific Inc., Waltham, MA, USA) containing a 1 mM EDTA buffer (pH = 8) at 95 °C for 20 minutes. Primary polyclonal antibody against Musashi-1 was then applied and incubated at 1:100 dilution for 16 h at 4 °C. Vimentin (clone V9) (1:100 dilution) was used as a positive control. A non-immunospecific IgG was used as negative control. All antibodies were obtained from Master Diagnóstica (Granada, Spain). The immunostaining was developed in an automatic immunostainer (Autostainer480S, Thermo Fisher Scientific Inc.) using a peroxidase-conjugated micropolymer and diaminobenzidine (Master Diagnóstica).

### Analysis of *MSI1* mRNA

Expression of *MSI1* mRNA in grafted bone normalized to native bone was also analyzed. In three of the abovementioned participants, one additional biopsy was collected from the same area, immediately submerged in Trizol reagent for the isolation of total RNA (Trizol^TM^ Plus RNA Purification kit, Invitrogen, Grand Island, NY) and frozen at −80 °C until further processing. Before RNA extraction, the samples were milled and then total RNA isolated following the manufacturer instructions. The reverse transcriptase-polymerase chain reaction (RT-PCR) was performed with an RT mix (PrimeScript™ RT Master Mix, Takara Bio Europe, Saint-Germain-en-Laye, France) using 1 µg of total RNA each for a final volume of 30 μL of complementary deoxyribonucleic acid (cDNA) using a thermal cycler. The primers used in the quantitative PCR using iQ-SYBR Green Supermix (Bio-Rad) are listed as Table 1. Evaluation of Periostin (gene *POSTN*) and runt-related transcription factor 2 (gene *RUNX2*) was performed to contextualized the role of *MSI1*. Each plate contained triplicates of the cDNA templates. The 2ddCt method was used to calculate gene expression levels relative to *GADPH*. Normalization was made to the native bone and data expressed as fold-change.

**Table 1:** Primer sequences for the mRNA evaluation of the specified genes.

Gene	Forward	Reverse
*GAPDH*	5-AGCTCATTTCCTGGTATGACAAC-3	5-TTACTCCTTGGAGGCCATGTG-3
*MSI1*	5-TGACCCTAAGGTGGCCTTCC-3	5-CGAGTCACCATCTTGGGCTG-3
*POSTN*	5-TTTCTACTGGAGGTGGAGAAAC-3	5-GTGACCTTGGTGACCTCTTC-3
*RUNX2*	5-ACCGTCTTCACAAATCCTCCC-3	5-AGCTTCTGTCTGTGCCTTCTG-3

### Culture of MSCs

Additional biopsies were collected from 3 participants. They were immediately placed in saline and quickly transfered to a flask containing DMEM (Sigma-Aldrich Co. LLC, St. Louis, MO, USA) supplemented with 10% fetal bovine serum (Gibco, Life Technologies Corporation, Grand Island, NY, USA) and 1% penicillin-streptomycin (Sigma-Aldrich Co. LLC). Bone biopsies were segmented into 1–2 mm pieces and left unaltered in an incubator at 37 °C, 5% CO_2_ until enough cells were estimated to be sufficient to be transferred to a bigger flask. Cells were detached by using a 0.25% trypsin solution (Sigma-Aldrich Co. LLC), always before reaching 80% confluence. Medium was changed every 48 h. At passage number 3, immunophenotype was established by detecting the classical MSCs markers by flow cytometry, anti-human antibodies CD11b, CD45, CD73, CD90 and CD105 (eBioscience, San Diego, CA, USA) in FACScantoII (Becton-Dickinson Biosciences, San Jose, CA, USA). Other cells at the same passage were centrifuged, fixed with 10% formalin for 24 h at 4 °C, transferred to 70% ethanol for 24 h and included in paraffin. MSI1 was detected as described above.

### Statistical analysis

According to previous studies from our group, bone grafted with a similar material as the one used in the current study showed an expression of MSI1 of 2.20 ± 0.83^23^. In the same study, the expression of MSI1 was approximately 50% lower when a lower osteogenic biomaterial was used. Thus, in native bone, a reduction to, at least, 30% can be expected. A group ratio of 0.5 was set because there might be more variability on the outcomes of the grafted bone than on the native bone. Thus, with an alpha of 0.05 and power of 80%, the calculated sample size was 37 in group 1 (grafted bone) and 18 in group 2 (non-grafted bone).

IBM SPSS-Windows 20.0 (SPSS Inc., Chicago, IL) was used for the analyses. Results are presented as mean (standard deviation) for continuous variables and as percentage(frequency) for categorical data. Chi-squared tests were used to compare categorical variables while Mann–Whitney *U* tests were used for continuous variables, since data were not normally distributed (p < 0.05, Shapiro-Wilk test). All tests were two-tail. Results were considered statistically significance when p values were below 0.05.

### Clinical Relevance

Detection of MSI1 in biopsies from grafted maxillary sinuses demonstrates the presence of multipotent mesenchymal cells. This marker could be used as predictor of remodeling activity and, eventually, as a therapeutic agent.

### Ethical aspects

All procedures performed in studies involving data from human participants were in accordance with the ethical standards of the institutional and/or national research committee and with the 1964 Helsinki declaration and its later amendments or comparable ethical standards.

This cross-sectional study was reviewed and approved by the Ethics Committee for Human Research of the University of Granada (307/CEIH/2017). Informed consent was obtained from every participant before any study procedure was initiated.

## Results

A total of 57 biopsies (39 from grafted and 18 from native bone) were obtained from 45 participants (28 men and 17 women) enrolled between 2013 and 2014, six months after the grafting procedure. Average age was 49 years (range 35–74). No significant differences were observed in the demographic variables age and gender between grafted and native sites (p > 0.05).

Morphological study (Table 2) of native bone showed normal cortical and trabecular bone (area = 42.2(21.6) %) with low remodeling activity. Medullary stroma (area = 57.8(14.3) %) was mainly composed by adipocytes with few fibroblasts, hematopoietic and lymphoid components. Grafted sites presented new trabecular bone (area = 36.5(15.1) %) over remnant particles (area = 25.5(17.5) %). Non-mineralized tissue (area = 38.0(15.3) %) was mainly constituted of fusiform cells in a highly dense and highly vascularized connective tissue. Some areas showed mature adipocytes in the medullary stroma.

**Table 2:** Morphological study of the different tissue compartments. Data is represented as mean percentage (standard deviation).

Tissue compartment	Native bone	Graft
Mineralized tissue	42.2(21.6)	36.5(15.1)
Non-mineralized tissue	57.8(14.3)	38.0(15.3)
Remnant graft particles	NA	25.5(17.5)

Nuclear or cytoplasmic immunohistochemical detection of MSI1 was observed in 97.4% (38/39) of biopsies from grafted sites, mainly in osteoprogenitor cells. Only 66.6% (12/18) of native bone biopsies showed immunopositivity for MSI1 and mainly in osteocytes. Immunopositivity in osteocytes was observed only at the nuclear level while in osteoblasts and osteoclasts it appeared in nuclei and cytoplasm. Two biopsies from grafted sites showed the Schneiderian membrane. In both cases, a low/moderate cytoplasmic MSI1 granular staining was observed. Immunopositivity of MSI1 in smooth muscle cells and endothelial cells was weak. No significant differences were observed between native and grafted bone. MSI1 was not detected in mature adipocytes.

**Figure 1:**
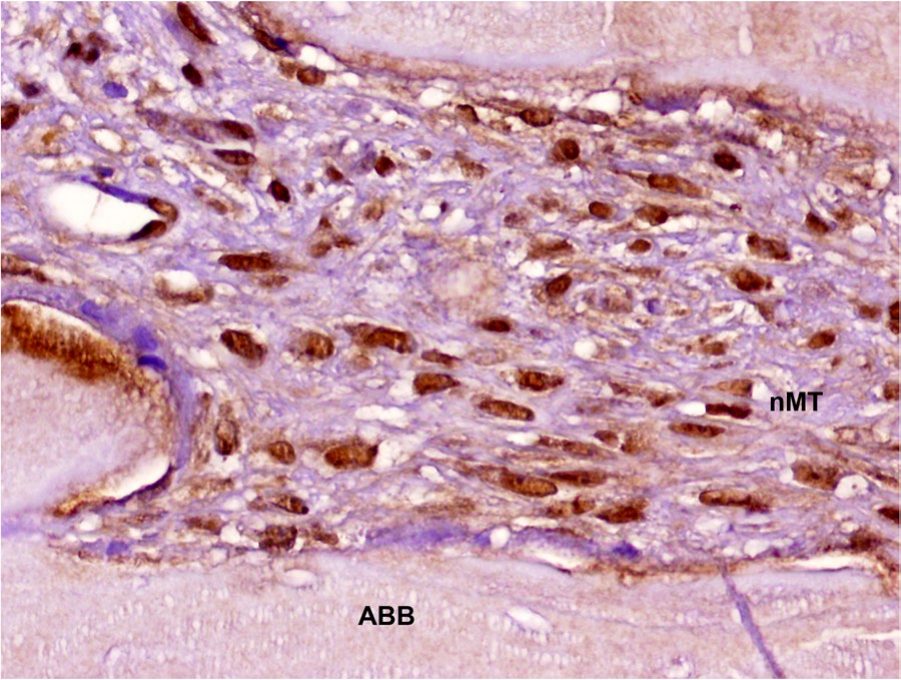
Detection of MSI1 in MSCs in non-mineralized tissue of a maxillary sinus floor elevation biopsy (peroxidase-conjugated micropolymer) (original magnification: 20x). ABB: anorganic bovine bone; nMT: non-mineralized tissue.

**Figure 2:**
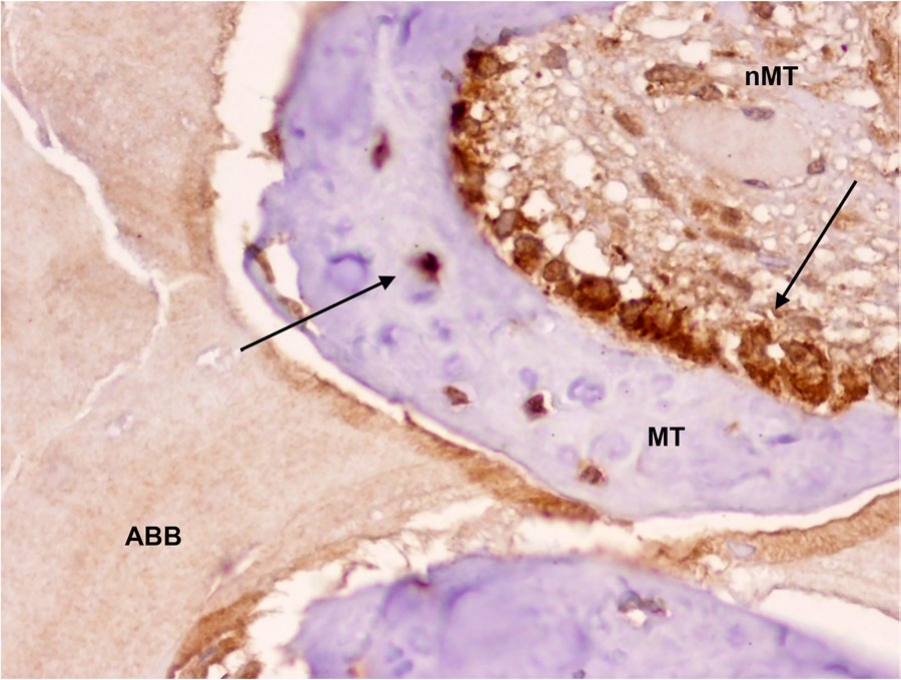
Detection of MSI1 in osteoblasts and osteocytes (arrows) and impregnation of anorganic bovine bone particle (black arrow) in a maxillary sinus floor elevation biopsy (peroxidase-conjugated micropolymer) (original magnification: 20x). ABB: anorganic bovine bone; nMT: non-mineralized tissue; MT: mineralized tissue.

**Figure 3:**
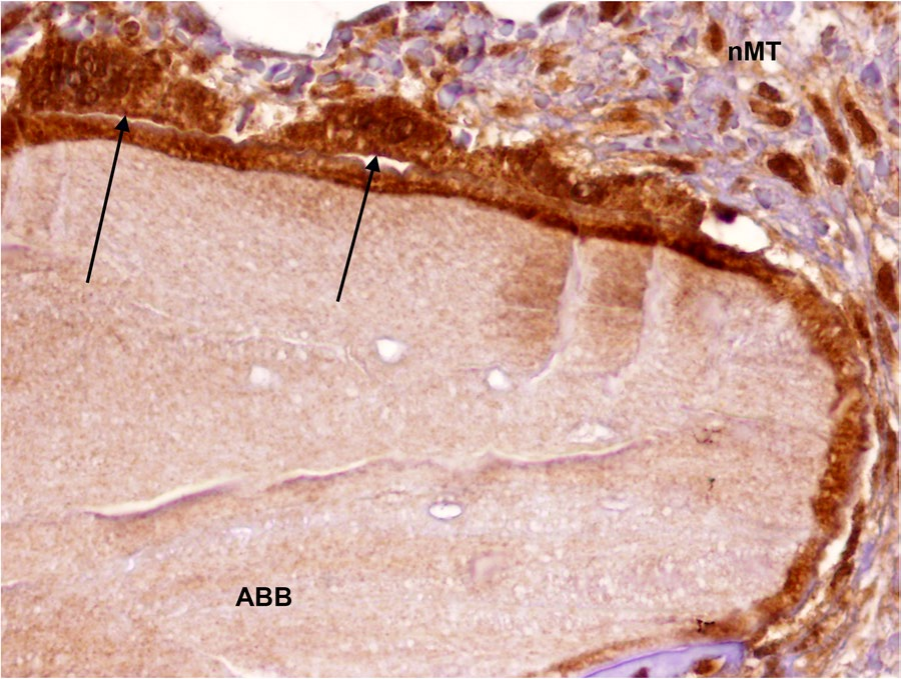
Detection of MSI1 in osteoclasts (black arrows) surrounding a particle of anorganic bovine bone of a maxillary sinus floor elevation biopsy (peroxidase-conjugated micropolymer) (original magnification: 20x). ABB: anorganic bovine bone; nMT: non-mineralized tissue.

Finally, mesenchymal cells of the non-mineralized compartment of native bone showed weak nuclear expression of MSI1. In contrast, high nuclear intensity was detected in fusiform cells in biopsies from grafted sites (0.28(0.13) vs. 2.10(0.14), respectively; p < 0.0001, Chi-squared test) (Table 3) (Fig. 1). In addition, detection of MSI1 in osteocytes, osteoblasts and osteoclasts was significantly higher in biopsies from grafted areas (Table 3) (Figs 2 and 3).

**Table 3:** Comparison of immunohistochemical detection of MSI1 in native bone vs. grafted bone after sinus floor elevation. Mean (Standard deviation) of a 0–3 scale. 0 = no staining; 1 = weak; 2 = moderate; 3 = intense.

Cells	Native bone	Graft	p value (Chi-squared test)
Osteocytes	0.89(0.67)	1.70(0.91)	0.001
Osteoblasts	0.56(0.92)	2.03(1.00)	0.000
Osteoclasts	0.22(0.54)	2.03(1.02)	0.000
Inflammatory infiltrate	0.56(0.70)	1.10(0.84)	0.020
Schneiderian membrane	0.11(0.47)	0.10(0.49)	0.936
Mesenchymal stromal cells	0.28(0.57)	2.10(0.92)	0.000
Smooth muscle cells	0.22(0.54)	0.23(0.53)	0.985
Endothelial cells	0.61(0.77)	0.58(0.59)	0.847

**Figure 4:**
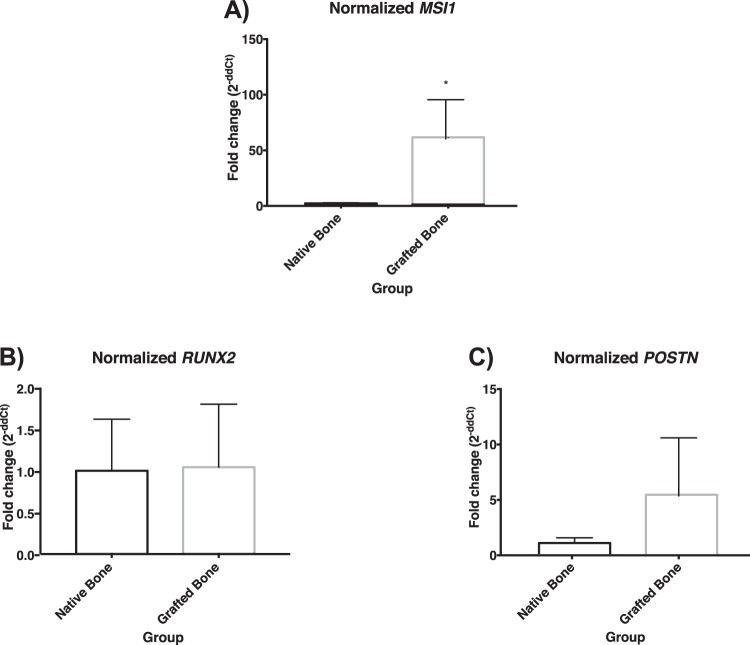
Normalized mRNA detection of (**A**) *MSI1*, (**B**) *POSTN* and (**C**) *RUNX2* in biopsies from native bone and grafted bone. *Statistically significant difference; p = 0.029.

In terms of mRNA expression, *MSI1* was significantly higher in grafted tissues (60.34(35.20)) than in native bone (1.00(0.51)) (p = 0.029, Mann–Whitney *U* test) (Fig. 4A). Although the expression of *POSTN* was higher in grafted bone (5.36(5.233)) than in native bone (1.00(0.47)), the difference was not statistically significant (p = 0.343, Mann–Whitney *U* test) (Fig. 4B). Finally, the expression of *RUNX2* was not significantly different in native bone (1.00(0.62)) than in grafted sites (1.04(0.76)) (p > 0.999, Mann–Whitney *U* test) (Fig. 4C).

**Figure 5:**
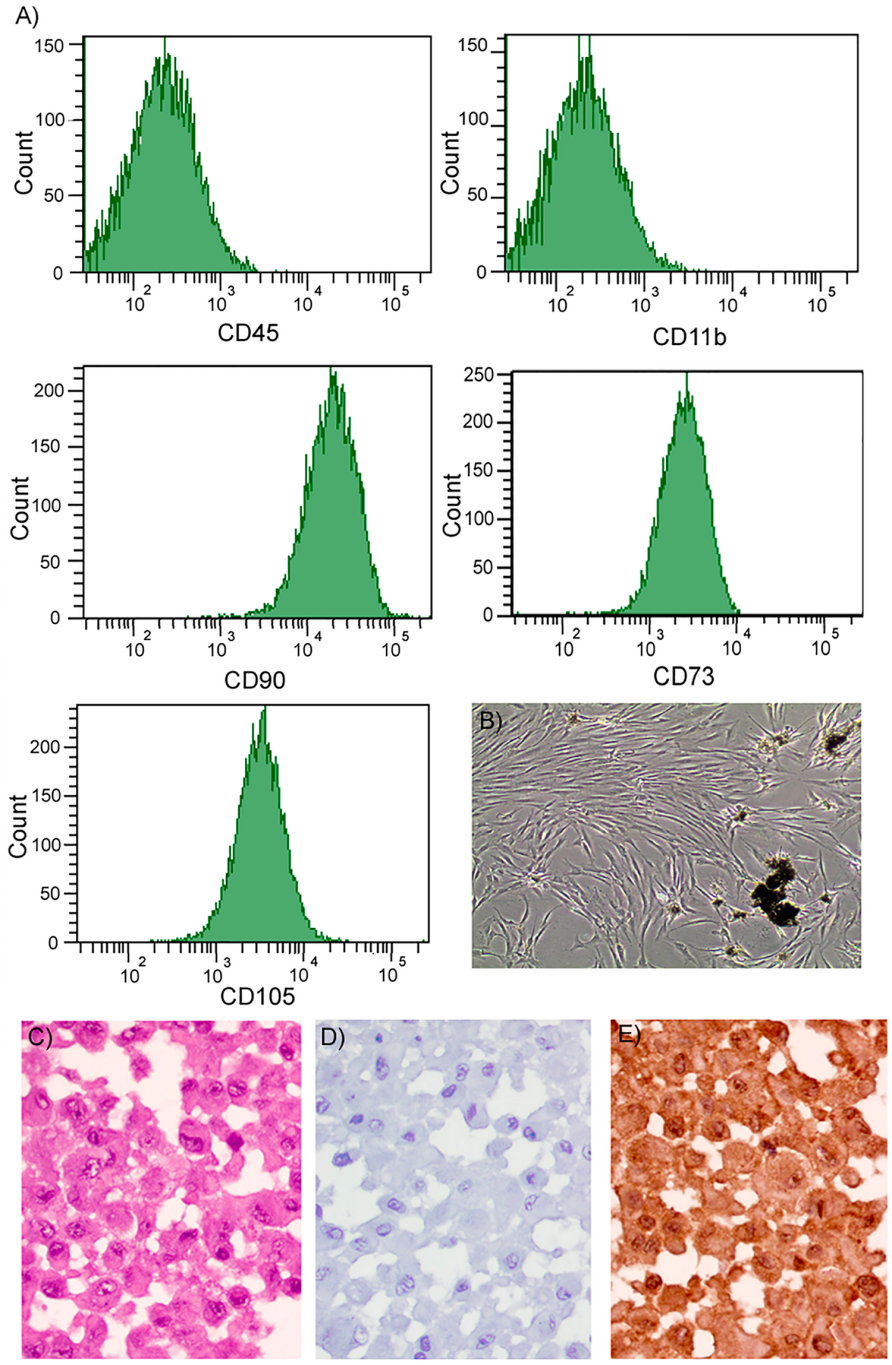
(**A**) Flow cytometry report representing negative detection of CD11b and CD45 and positive detection of CD73, CD90 and CD105. (**B**) Representative image of cultured cells at initial passages with remnants of mineralized tissue. (**C**) H&E staining of a pellet of cultured of adherent cells showing the integrity of the cells. (**D**) Isotype control. (**E**) Nuclear and cytoplasmic detection of MSI1 (peroxidase-conjugated micropolymer) (original magnification: 40x).

Flow cytometry analysis of cultured adherent cells at their passage number 3 reflected high positivity for CD73, CD90 and CD105 but was negative for CD11b and CD45 (Fig. 5A). 95% of cultured cells were immunopositive for MSI1 in their nucleus and cytoplasm (Fig. 5B–E).

## Discussion

In this study, to our knowledge, the higher detection of MSI1 and expression of its mRNA in newly formed bone 6 months after bone grafting procedures is demonstrated for the first time in literature in comparison to data from non-grafted native bone. Our group has previously demonstrated a higher expression of MSI1 in MSCs in biopsies collected from sites grafted with the same mix used in the current study (ACB + ABB: 2.20(0.83)) in comparison to those grafted with a different graft (ACB + freezed-dried bone allograft: 0.80(0.75))^23^. These findings, together with a higher number of osteoid lines, blood vessels and overall cellularity in the ACB + ABB group, were referred to be representative of an increased remodeling activity. In the current study, even lower MSI1 was found in MSCs in native bone (0.28(0.57)) than in ACB + freezed-dried bone allograft mix. However, a similar detection intensity was observed in the ACB + ABB group (2.10(0.92)). Thus, it could be confirmed that MSI1 is representative of bone remodeling activity and its expression is low in native bone, higher when using allografts together with autogenous grafts and even higher if the graft mix includes anorganic bovine bone.

Sinus floor elevation and grafting is a highly predictable technique. However, bone graft maturation is influenced by several variables, such as type of edentulism, history of periodontal disease, habits such as tobacco and alcohol consumption^24^, the presence of teeth adjacent to the maxillary sinus, thickness of the lateral wall of the sinus, as well as participant’s age and gender^25^. In this context, it must be highlighted that, when studying bone biomaterials, not only a radiographic or volumetric analysis should be performed. It is of extreme importance to analyze what is happening at the biological level as the only way to understand the specific response that the biomaterial is inducing. This is also the only way to propose improvements.

MSCs are of high interest in regeneration procedures in the maxillofacial area^26^. Self-renewal and ability to differentiate into multiple cell types, including osteogenic, adipogenic, chondrogenic or myogenic lineages, are key characteristics of MSCs^27^. MSCs are, therefore, key elements in bone graft maturation and osseointegration processes used in implant dentistry. Because of this, they have been used in bone regenerative procedures, including sinus floor elevation^28^. The expression of MSI1 by MSCs have been previously reported in those differentiating into neuroglial cells, including MSCs from the Wharton jelly in the umbilical cord^16^. In bone, fusiform cells could represent either MSCs or fibroblasts; however, their expression of MSI1 confirm their MSC nature. Moreover, cell cultures obtained by explantation (with no digestion, supplementation or differentiation stimulants) demonstrated the positive surface expression of the triad CD73/CD90/CD105, i.e., surface markers of MSCs. Similar populations from native bone have been previously obtained with the same methodology, although no MSI1 expression was studied^29^.

MSI1 acts in cell differentiation through the *Notch* pathway^14^. In bone, the target of MSI1 would be the *Wnt* gene that regulates bone remodeling by favoring cell proliferation, differentiation or apoptosis of undesired cells^30^. Un-inhibiting MSCs differentiation in an osteogenic environment would ultimately result in the proliferation of osteoblastic cells and the induction of bone formation^31^. In this study, the expression of other bone-related markers, such as *POSTN* and *RUNX2*, was studied in order to contextualized the role of *MSI1*. In this case, the differences between the expression of *POSTN* and *RUNX2* in native bone vs. grafted bone were not statistically significant. This could be explained by a fast healing in the grafted sites that after 6 months have completed the reparative processes. Still, after that time, the area can be described as a more osteogenic differentiating environment compared to native bone. In fact, the expression of MSI1, POSTN and RUNX2 has been demonstrated to be correlated with the osteogenic process in a fracture healing model. Interestingly, this correlation was not found in mature chondrocytes, which highlights the importance of MSI1 in bone differentiation (manuscript under evaluation). Not surprisingly then, in the current study, there was a statistically significant higher detection of MSI1 in bone-related cells, such as osteoclasts, osteoblasts, osteocytes and MSCs. However, the detection of MSI1 in other cell types, such as smooth muscle and endothelial cells, was not different from native bone and grafted bone. This reflects a non-determinant role in those vascular structures.

Therefore, the activation of these signaling pathways, among others, and together with the stimulus generated by the biomaterial surface and chemical characteristics, could ultimately induce a faster bone healing around particular bone grafts. *Ex vivo* stimulation strategies before re-implantation could also be proposed^32^. It is also important to note that MSCs from different origins may show different functionalities^33^, which in fact highlights the importance of studying novel markers of MSCs from bone marrow origins and/or with the potential to differentiate to osteogenic precursors. This will ultimately increase our understanding of the bone regeneration/remodeling processes so that new regenerative strategies can be proposed in the future. In the current study, MSI1 was detected in osteoprogenitor cells in 97.4% of biopsies from grafted sites vs. 66.6% of biopsies from native bone. Although MSI1 was detected in osteoblasts, osteoclasts and osteocytes, its specific function in these cell populations is still unknown.

In the current study, we have limited our analysis to only one biomaterial used for bone graft, which could potentially modify the expression of MSI1. The response to other biomaterials should also be analyzed. In addition, the group umbalance was set considering that native bone would be less variable than grafted bone, which in the end requires a response-to-injury type of reaction. This might be influenced by a high number of different factors. The status of native bone might be too. So, further evaluation of potential patient-dependent factors influencing the expression of MSI1 should also be analyzed in future studies.

## Conclusion

Bone repair and regeneration is mainly driven by MSCs. MSI1 was detected in osteoprogenitor and mesenchymal cells. The intensity and levels of mRNA were significantly higher in biopsies obtained from grafted bone in comparison to biopsies obtained from native bone, which reflects its importance in the remodeling/repair events that occur after maxillary sinus floor elevation.
